# Early seizure freedom with [ ^18^F]fluorodopa positron emission tomography response after isocitrate dehydrogenase inhibition with vorasidenib: First case report

**DOI:** 10.1111/epi.18593

**Published:** 2025-08-06

**Authors:** Roberta Rudà, Francesco Bruno, Alessia Pellerino, Edoardo Pronello, Michela Zotta, Silvia Morbelli

**Affiliations:** ^1^ Division of Neuro‐Oncology, Department of Neuroscience “Rita Levi Montalcini” University and City of Health and Science Hospital, University of Turin Turin Italy; ^2^ Division of Nuclear Medicine, Department of Medical Sciences University and City of Health and Science Hospital, University of Turin Turin Italy

**Keywords:** amino acid PET, F‐DOPA PET, seizures, vorasidenib

## Abstract

Vorasidenib, a dual isocitrate dehydrogenase 1/2 (IDH1/2) inhibitor, showed superior efficacy in prolonging progression‐free survival and time to next intervention in *IDH*‐mutant grade 2 gliomas. This case is part of an ongoing institutional study exploring the impact of vorasidenib on seizure control and the potential of [^18^F]fluorodopa (F‐DOPA) positron emission tomography (PET) to detect treatment response earlier than magnetic resonance imaging (MRI). A 52‐year‐old patient with grade 2 *IDH*‐mutant 1p19q‐codeleted oligodendroglioma and persistent postoperative seizures received vorasidenib. He achieved early seizure freedom from the first month of therapy without any change of antiseizure medication (ASM). At 3 and 6 months after treatment, MRI showed stable disease (with a slight progressive reduction in tumor volume), whereas F‐DOPA PET revealed a significant decrease in tracer uptake starting from the third month, which was confirmed at 6 months, corresponding to a partial response according to PET Response Assessment in Neuro‐Oncology 1.0 criteria. This is the first report of an *IDH*‐mutant grade 2 glioma patient achieving early seizure control and metabolic response on F‐DOPA PET after vorasidenib. It highlights the potential of vorasidenib for seizure management and the value of F‐DOPA PET for early treatment assessment. Further studies are required to evaluate long‐term seizure control and potential reduction of ASM in *IDH*‐mutant low‐grade gliomas treated with vorasidenib.

## 1 INTRODUCTION

In the INDIGO trial, the dual isocitrate dehydrogenase 1/2 (IDH1/2) inhibitor vorasidenib demonstrated superior efficacy over placebo in prolonging magnetic resonance imaging (MRI)‐based progression‐free survival and time to next intervention in patients with *IDH1/2*‐mutant grade 2 glioma after surgery.^1^ Therefore, vorasidenib received rapid US Food and Drug Administration approval in August 2024 as the first targeted therapy for *IDH1/2*‐mutant grade 2 gliomas. Although the European Medicines Agency approval process is still underway, a compassionate use program for vorasidenib is already ongoing in many European countries. With the introduction of vorasidenib into clinical practice, new challenges and questions are emerging. First, given that D2‐hydroxyglutarate, the metabolic product of *IDH*‐mutant enzymes, plays a key role in epileptogenesis by mimicking the excitatory neurotransmitter glutamate, the potential of vorasidenib to improve seizure control is attractive.^2^, ^3^ Second, because conventional MRI may not be able to detect subtle changes in gliomas following treatment with antineoplastic drugs, amino acid positron emission tomography (PET) could provide earlier insights into treatment response.^4^

Here, we describe the case of a patient diagnosed with a grade 2 *IDH*‐mutant 1p19q‐codeleted oligodendroglioma, with persistent seizures after surgery, who received vorasidenib alone and achieved early seizure freedom coupled with a metabolic PET response.

## 2 MATERIALS AND METHODS

The evaluation of seizure semiology and outcome was performed according to the 2025 Updated Classification of the Epileptic Seizures of the International League Against Epilepsy (ILAE).^5^ Seizure frequency was calculated as the number of seizure days from the last visit based on a patient diary that was reviewed at each monthly clinical visit. Seizure freedom was defined as the complete absence of seizures or auras according to ILAE outcome classification for seizures.

MRI response to vorasidenib was evaluated according to Response Assessment in Neuro‐Oncology (RANO) criteria for low‐grade gliomas^6^ and [^18^F]fluorodopa (F‐DOPA) PET response according to PET RANO criteria 1.0^7^ at 3 and 6 months. In accordance with PET RANO recommendations, a threshold of 1.6 times the mean background activity was applied for standardized assessment to define the metabolic tumor volume (MTV). A visual plausibility check, based on offline fused PET/MRI images, was also performed to ensure accurate segmentation—particularly to exclude physiologically high‐uptake regions, such as the striatum.

## 3 RESULTS

A 52‐year‐old male had no significant medical history experienced since July 2022 episodes of epigastric aura, left‐hand paresthesias, and impaired awareness. These episodes, initially sporadic, progressively became more frequent, up to once daily. In April 2024, the patient underwent an initial neurological evaluation. Due to suspected nonmotor focal temporal seizures, electroencephalography (EEG) along with brain MRI with gadolinium contrast was recommended. The EEG showed the presence of focal slow theta activity in the right frontal region, enhanced by hyperventilation, and the MRI revealed a right frontotemporoinsular T2/fluid‐attenuated inversion recovery (FLAIR)‐hyperintense, non‐contrast‐enhancing lesion. Therefore, antiseizure medication (ASM) with levetiracetam 1000 mg twice daily was started, and in May 2024 the patient underwent a subtotal resection. The diagnosis was grade 2 *IDH1* R132H mutant, 1p/19q codeleted oligodendroglioma with the following histomolecular characteristics: Ki‐67 < 5%, absence of mitoses, necrosis, or microvascular proliferations, *pTERT* mutant, and *CDKN2A/B* intact.

**FIGURE 1:**
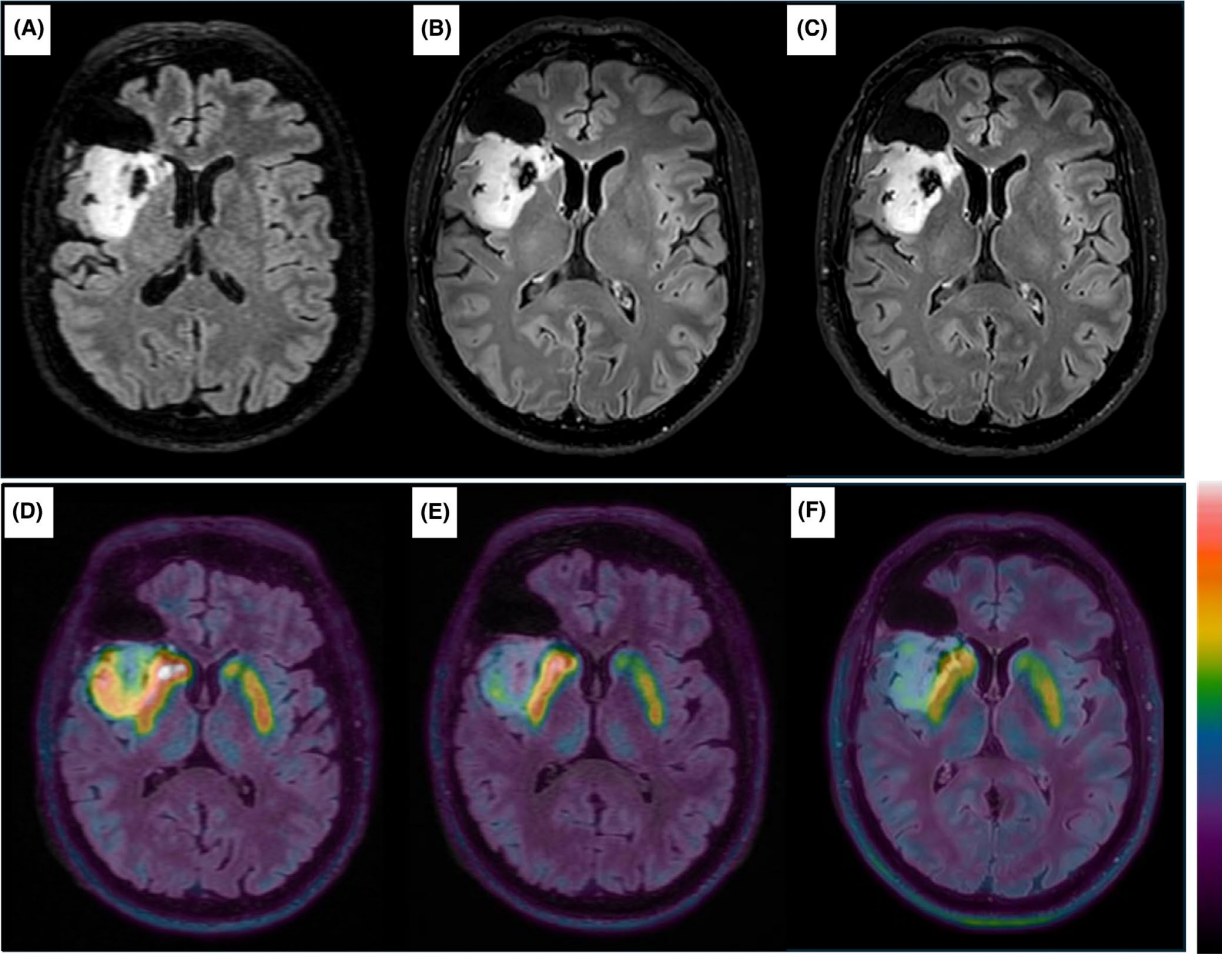
Brain magnetic resonance imaging (MRI) T2/fluid‐attenuated inversion recovery three‐dimensional axial sequences and [^18^F]fluorodopa (F‐DOPA) positron emission tomography (PET) at baseline and after three cycles of vorasidenib. (A) MRI at baseline. (B) MRI after three cycles. (C) MRI after six cycles. (D) [^18^F]‐DOPA PET at baseline. (E) [^18^F]‐DOPA PET after three cycles. (F) [^18^F]‐DOPA PET after six cycles. In PET images, for all timepoints, the maximum uptake of the scale, as presented in the color bar (white), was placed in the region of maximum uptake.

**FIGURE 2:**
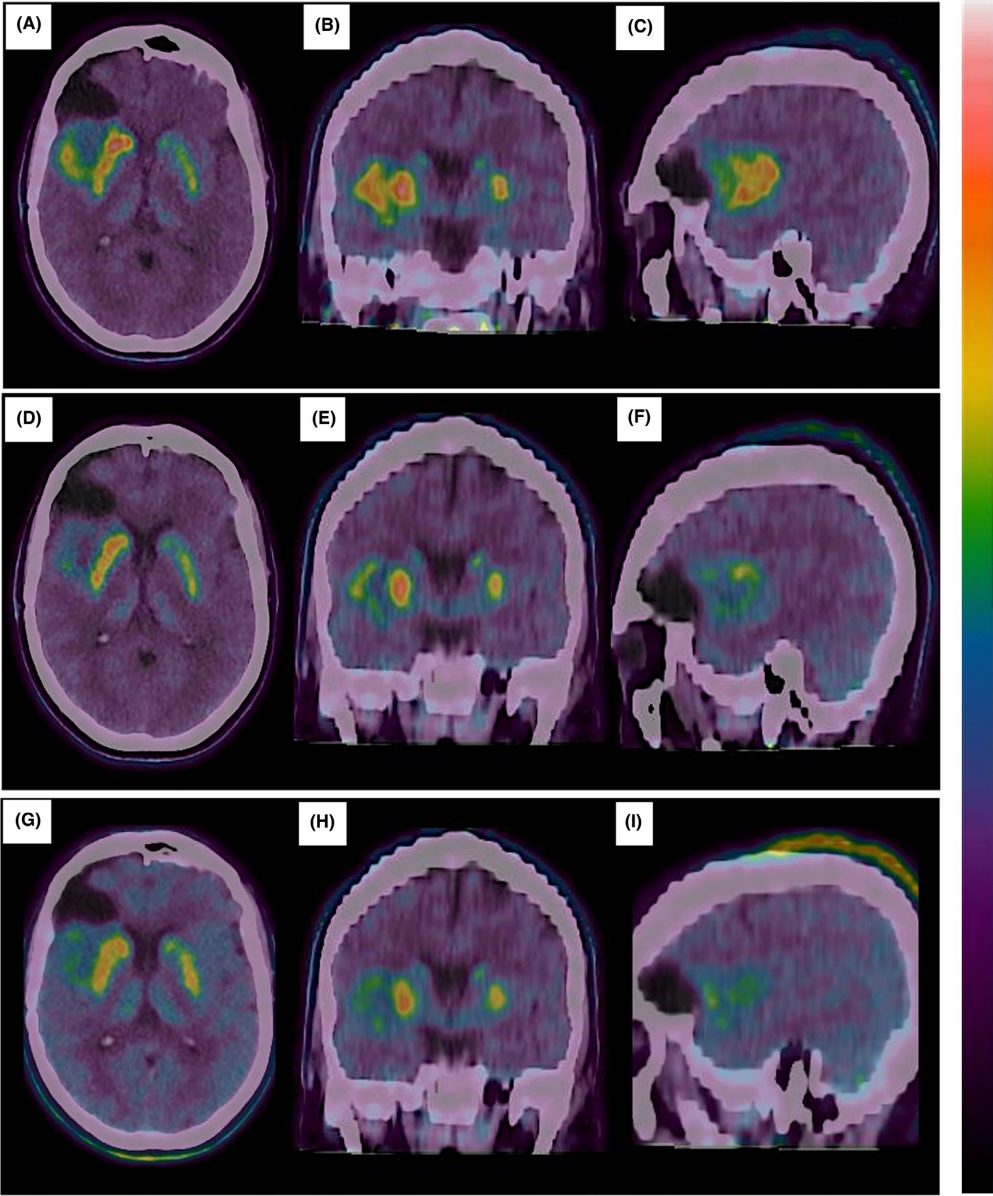
[^18^F]Fluorodopa positron emission tomography at baseline and after three and six cycles of vorasidenib. (A) Baseline, axial. (B) Baseline, coronal. (C) Baseline, sagittal. (D) After three cycles, axial. (E) After three cycles, coronal. (F) After three cycles, sagittal. (G) After six cycles, axial. (H) After six cycles, coronal. (I) After six cycles, sagittal. For all timepoints, the maximum uptake of the scale, as presented in the color bar (white), was placed in the region of maximum uptake.

The patient did not develop any new neurological symptoms following surgery but continued to experience focal seizures with unchanged semiology at a frequency of approximately two per week, despite ongoing treatment with levetiracetam (a second ASM was not introduced, in accordance with the patient's preference). Three months after surgery (September 2024), the MRI showed a 32.2‐cm^3^ nonenhancing residual tumor in the right insula on three‐dimensional (3D) T2/FLAIR sequences (Figure 1A). A PET scan with F‐DOPA revealed increased tracer uptake in the tumor (maximum standardized uptake value [SUVmax] = 3.2, maximum target to background [TBRmax] = 2.5, MTV = 16.3 cm^3^; Figures 1D and 2A–C). After discussion by the Multidisciplinary Tumor Board, the patient was enrolled in an Expanded Access Program for vorasidenib (local ethics committee protocol: 76/2024). Due to the absence of neurological symptoms and edema on MRI, corticosteroid therapy was never used.

Vorasidenib 40 mg daily was started in October 2024, and early seizure freedom after 1 month of treatment was achieved without any change of ASM. Now (June 2025) the patient has completed seven cycles of vorasidenib, with the eighth currently ongoing. MRI after three cycles (January 2025) and after six cycles (April 2025) showed stable disease per RANO criteria for low‐grade gliomas,^6^ with a slight progressive but nonsignificant volume reduction on 3D‐FLAIR sequences (32.2 cm^3^ at baseline, Figure 1A; 31.5 cm^3^ after three cycles, Figure 1B; 28.1 cm^3^ after six cycles, Figure 1C). In contrast, F‐DOPA PET showed a significant reduction of the MTV, indicating a partial response according to PET RANO 1.0 criteria after three cycles (SUVmax = 3.2, TBRmax = 2.3, MTV = 8 cm^3^; Figures 1E and 2D–F) and six cycles (SUVmax = 2.5, TBRmax = 2.3, MTV = 4 cm^3^; Figures 1F and 2G–I). The patient had excellent adherence to the treatment, did not report any adverse event, and had normal liver enzyme levels. He is still seizure‐free at 8 months from start of vorasidenib.

## 4 DISCUSSION

To our knowledge, this is the first report of a grade 2 glioma patient with persistent seizures after surgery, who achieved early seizure freedom following the dual *IDH*‐mutant inhibitor vorasidenib. The positive impact of radiation and chemotherapy (procarbazine–lomustine–vincristine, temozolomide) on seizures in low‐grade gliomas, in terms either of seizure reduction ≥ 50% or seizure freedom, is well known.^8^ A recent study with evaluation of seizures over the whole disease trajectory of grade 2 and 3 *IDH*‐mutant gliomas has reported that seizure freedom for 6 months following adjuvant treatments is predictive of longer progression‐free survival, that is, more durable tumor control.^9^ Conversely, there is a lack of information on the outcome of seizures following treatment with vorasidenib, as in the INDIGO trial uncontrolled seizures were excluded from enrollment. Long‐term studies are needed to evaluate the magnitude and duration of seizure control and the possibility to reduce or withdraw ASMs in subjects with *IDH*‐mutant gliomas who respond to vorasidenib; in this regard, the rate of seizure control following standard radio‐ and chemotherapy will serve as a benchmark for the evaluation of the role of IDH inhibitors.

Our case highlights the importance of seizures as a marker of tumor activity in *IDH*‐mutant grade 2 gliomas and seizure control as a secondary endpoint of efficacy in clinical trials.^10^ Also, it supports the value of amino acid PET as a sensitive tool for assessing treatment efficacy, offering earlier information than conventional or volumetric MRI. In this regard, the present case extends to F‐DOPA PET imaging the preliminary evidence provided by Galldiks et al. on the capability of PET with [^18^F]fluoroethyltyrosine to allow earlier evaluation of functional changes following vorasidenib.^11^ Interestingly, a case report has described increased F‐DOPA PET uptake in both tumor and peritumoral brain parenchyma of an epileptogenic diffuse astrocytoma in a child.^12^ Together with our case, these findings may suggest that F‐DOPA PET could capture seizure activity; confirmation in larger well‐designed studies is needed.

A deeper understanding of the mechanisms of seizure reduction following IDH inhibitors by employing experimental models of *IDH*‐mutant gliomas is needed,^13^ as, so far, there is lack of information. It has been reported that 2‐HG is exported into the microenvironment, and the excitatory effects on neurons are rapid and do not seem to depend on a direct action on ionotropic glutamate receptors.^2^ 2‐HG could also be able to activate mTOR signaling.^14^, ^15^

Due to the importance of 2‐HG in epileptogenesis and tumor growth of *IDH*‐mutant gliomas, the evaluation of usefulness of serial monitoring of the blood level of 2‐HG in parallel with tumor and seizure changes following vorasidenib is important, and it is ongoing at our institution.

Given the encouraging response and good tolerability with vorasidenib, we are continuing the treatment. Moreover, based on early evidence of seizure improvement following IDH inhibition, we anticipate sustained seizure control as long as the patient maintains response on MRI and amino acid PET.

Several questions remain open. Does seizure improvement following IDH inhibition allow ASM tapering in selected patients? What is the risk of seizure recurrence after treatment interruption? Might IDH inhibition enhance seizure control also in patients with refractory epilepsy? Future trials in patients with *IDH*‐mutant gliomas of different grades of malignancy (2–4) should investigate the efficacy of IDH inhibitors on pharmacoresistant seizures and validate the additional value of PET monitoring with amino acid tracers in evaluating both tumor and seizure response over the entire disease trajectory (before and after surgery and following chemotherapy and targeted agents).

## AUTHOR CONTRIBUTIONS

*Conceptualization:* Roberta Rudà and Francesco Bruno. *Writing:* Roberta Rudà, Francesco Bruno, Alessia Pellerino, Edoardo Pronello, Michela Zotta, and Silvia Morbelli. *Final draft revision:* Roberta Rudà and Francesco Bruno.

## CONFLICT OF INTEREST STATEMENT

None of the authors has any conflict of interest to disclose relevant to this research activity. We confirm that we have read the Journal's position on issues involved in ethical publication and affirm that this report is consistent with those guidelines.

## PATIENT CONSENT STATEMENT

The authors obtained written informed consent from the patient described in this article.
